# HIV, progestins, genital epithelial barrier function, and the burden of objectivity^†^

**DOI:** 10.1093/biolre/ioaa078

**Published:** 2020-05-15

**Authors:** Rodolfo D Vicetti Miguel, Nirk E Quispe Calla, Thomas L Cherpes

**Affiliations:** Department of Comparative Medicine, Stanford University School of Medicine, Stanford, CA, USA

**Keywords:** genital epithelial barrier function, HIV, progestins

## Abstract

Contributions from a diverse set of scientific disciplines will be needed to help individuals make fully informed decisions regarding contraceptive choices least likely to promote HIV susceptibility. This commentary recaps contrasting interpretations of results from the Evidence for Contraceptive Options and HIV Outcomes (ECHO) Trial, a study that compared HIV risk in women using the progestin-only injectable contraceptive depot medroxyprogesterone acetate (DMPA) vs. two other contraceptive choices. It also summarizes results from basic and translational research that establish biological plausibility for earlier clinical studies that identified enhanced HIV susceptibility in women using DMPA.

## Teaser

Basic and translational research studies provide strong indication that the contraceptive depot medroxyprogesterone acetate weakens genital mucosal barrier function, a vital first-line defense against HIV and other sexually transmitted pathogens.

## A call for clarity

Globally, most new HIV infections occur in sub-Saharan Africa (SSA), with women < 25 years of age the regional cohort most vulnerable to infection [1]. In SSA, the progestin-only injectables depot medroxyprogesterone acetate (DMPA) and norethindrone acetate (NET-EN) are used by ~15 million women (i.e., nearly half of all regional contraceptors) [2, 3]. This level of usage differs from other developing areas that show greater proportional reliance on oral contraception, sterilization, and intrauterine devices for contraception [4]. Moreover, the distinct mix of large HIV burden, cohort of young women especially vulnerable to HIV, and sizeable reliance on injectable progestins in SSA has sparked an ongoing debate regarding DMPA use, a controversy particularly fueled by results from multiple observational studies that indicated DMPA enhances HIV susceptibility [5]. Although results from these studies have been mixed, a 2016 systematic review of all higher quality studies estimated risk of HIV acquisition increases ~40% in women using DMPA vs. no hormonal contraception (HC) [6].

It is important to acknowledge, however, that identification of DMPA as a significant HIV risk factor in these observational studies may have been erroneously created by a higher frequency of unprotected sex in women using DMPA vs. no HC [7]. Although use of a randomized clinical trial would not eliminate the possibility that women initiating contraceptive use engage in higher risk activities than non-contraceptors or prevent study participants from switching contraceptives, it was considered the type of study best able to prevent behavioral confounders from masking the precise relationships between DMPA and HIV susceptibility and the biological effects of DMPA on antivirus host defense and HIV transmission [8]. Based on this possibility, the Evidence for Contraceptive Options and HIV Outcomes (ECHO) Trial Consortium was organized to compare HIV acquisition in women from eSwatini, South Africa, Kenya, and Zambia randomized in a 1:1:1 fashion to initiate use of DMPA, copper intrauterine device (Cu-IUD), or levonorgestrel (LNG) implant.

Published in June 2019, the ECHO Trial Consortium reported that when compared to one another, these contraceptives were not statistically significant HIV risk factors [9]. About 1 month after the ECHO Trial results were made available, the World Health Organization (WHO) assembled a cadre of family planning experts, clinicians, epidemiologists, guideline methodologists, program managers, and policy makers to review medical eligibility criteria for contraceptive use, particularly for women at high risk of HIV infection. The gathered Guideline Development Group (GDG) resolved that findings from the ECHO Trial superseded all previously acquired observational data and concluded that there was no need to restrict DMPA use. Their recommendations were disseminated to the public in August 2019 [10].

## One ECHO, alternative views

Soon after these recommendations were publicly disseminated, an alternative interpretation of the ECHO Trial data was voiced [11]. Rationale for this opposing view emanated from the decision of ECHO Trial investigators to define relative risk of HIV in women using DMPA, Cu-IUD, or LNG implant and the similarly high HIV incidence in these three study groups (DMPA: 4.2/100 woman years; Cu-IUD: 3.9/100 woman years; and LNG implant: 3.3/100 woman years) [9]. Specifically, as effects of using Cu-IUD or LNG implants on HIV susceptibility are undefined, it was argued that to conclude comparable rates of HIV acquisition in the ECHO Trial supports unrestricted DMPA access risks an overly broad interpretation of study results [11]. Moreover, while observational studies indicated HIV risk increased ~40% in women using DMPA, it was argued that much smaller increases in risk can adversely impact women in areas of the world with high burden of HIV disease [11, 12]. This possibility acquired specific relevance when interpretating ECHO Trial data, a study that saw women using DMPA ~29% more likely to acquire HIV than women using Cu-IUD (*P*-value = 0.06) [9]. Inability of this between-group comparison to achieve statistical significance (i.e., display a *P*-value ≤ 0.05) must therefore be reconciled with realization that the ECHO Trial had been powered to identify statistically significant differences in HIV risk, which were >50% [9, 11]. Whereas the ECHO Trial Consortium published an erratum to their original report to acknowledge even relatively small effects on HIV risk can influence public health policymaking related to contraceptive use and HIV prevention [13], this concession played an unknown role in the decision of the GDG to recommend unrestricted access to DMPA, including for women at high risk of HIV infection [10].

Playing a more explicit role in the GDG decision to recommend that women be uniformly eligible to use DMPA (and all other progestin-only contraceptives) was their opinion of previously acquired data that explored biological plausibility for altered HIV susceptibility in women using these contraceptives. As stated in their executive summary, the GDG opined that the sparse and contradictory data made it unclear if any of the explored biological mechanisms were clinically relevant [10]. However, as unrestricted DMPA access may affect both an individual woman’s HIV risk and efforts to curb the HIV pandemic, the current commentary will summarize experimental and clinical findings that show DMPA and other exogenous progestins weaken genital epithelial barrier function, an essential antivirus host defense. Our commentary will also provide evidence that the accumulated data defining this progestin-mediated weakening of the genital epithelial barrier is neither scant nor inconsistent.

## Epithelial barriers: the basics

All mucosal surfaces, including in the female genital tract, are lined by epithelial cells whose borders are occupied by junctional complexes restricting paracellular migration of microorganisms and other luminal contents [14]. These intercellular junctions are termed tight junctions (zonula occludens), adherens junctions (zonula adherens), and desmosomes (macula adherens) [15]. Although all three complexes promote cell–cell adhesion, desmosomes specifically confer mechanical tissue strength by anchoring a network of flexible intermediate filaments to plasma membrane [16]. The desmosomal interaction with intermediate filaments increases the resistance of epithelial tissue to mechanical insult and promotes epithelial integrity. The core protein components of the desmosome include the cadherins desmogleins (Dsgs) and desmocollins (Dscs), desmoplakin, and armadillo proteins such as plakoglobulin and plakophilins [17, 18]. These components are not uniformly expressed throughout an epithelial layer, and regulation of this process serves to increase plasticity in basal proliferative layers and strength and barrier function in more superficial layers [19]. For example, expression of desmoglein 2 (Dsg2) is basal layer concentrated while the expression of Dsg2 and desmocollin 1 (Dsc1) becomes progressively more prominent as epithelial cells migrate toward the lumen surface [16]. As static contact between cells are unable to be conserved during differentiation, remodeling, and repair of epithelial tissue, desmosomal protein expression is regulated at the transcriptional level and by activity of various proteases including the kallikrein family of serine proteases [20, 21]. Factors that alter expression of these molecules thus have capacity to modulate normal desmosome composition and promote epithelial barrier dysfunction. In fact, individuals genetically deficient in DSG1 display profound disruption of epithelial barrier function.

**Figure 1:**
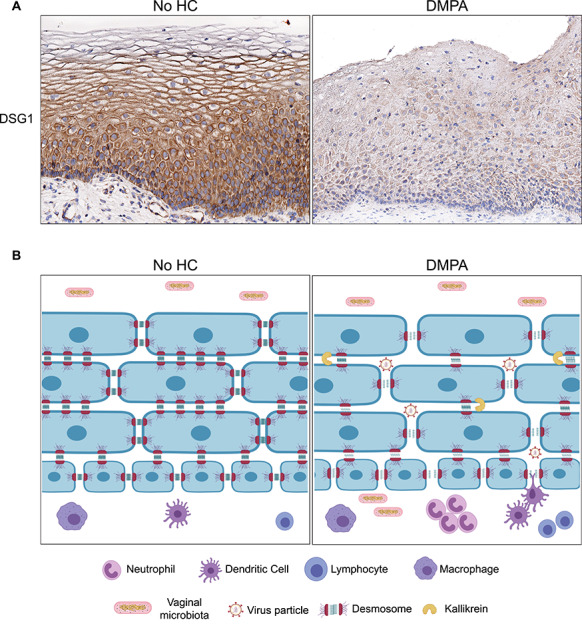
DMPA decreases expression of genital desmosomal proteins and weakens genital epithelial barrier function. (A) Immunohistochemical staining of vaginal biopsy tissue obtained from a woman using no form of HC and 1 month after she elected to initiate DMPA use shows that DMPA reduces levels of the cell–cell adhesion molecule DSG1. (B) Cartoon depicts a scenario by which DMPA-mediated loss of desmosomal proteins, including DSG1, weakens genital epithelial barrier function and increases HIV susceptibility. As suggested by animal model data, desmosomal protein loss is sequelae to DMPA-mediated downregulation of desmosomal gene expression and increased kallikrein activity. The compromised genital epithelial barrier function increases genital inflammation by enhancing vaginal microbiota entry into submucosal tissue and promotes infection by facilitating virus particle invasiveness and interaction with host target cells.

## HIV, progestins, and barrier function: an evidence-based case for biological plausibility

Although mechanisms underlying the effect were not defined, DMPA has long been used experimentally to make laboratory animals uniformly susceptible to a variety of genital bacterial and viral pathogens [22–24]. Eo ipso this offered biological plausibility for clinical studies identifying DMPA as a significant HIV risk factor but did not provide mechanistic insights into this relationship. Conversely, Marx et al. [25] showed >20 years ago that exogenous progesterone administration to rhesus macaques (RMs) thinned vaginal epithelial tissue and increased susceptibility to genital simian immunodeficiency virus (SIV) infection. They further demonstrated that treating ovariectomized RM with exogenous estrogen (E) increased vaginal epithelial thickness and keratinization and protected against vaginal SIV infection [26, 27]. Together, these studies provided early indication for the capacity of exogenous sex steroids to regulate genital epithelial barrier function.

In a 2012 report, Hope et al. [28] used untreated RM to show that expression of desmoglein 1/2 (the antibody used in this publication did not discriminate between DSG1 and DSG2) was concentrated in superficial epithelium layers of vaginal squamous epithelium and endocervical columnar epithelium. These investigators also identified comparable patterns of desmoglein 1/2 expression in genital tissue from women. However, neither the nonhuman primate nor human data quantitatively assessed Dsg expression or explored functional implications of histologic findings [28]. This research group also used human cervical explants and vaginal HIV-1 inoculation of untreated RM to show that depth of virus penetration into squamous epithelium correlated directly with tissue permeability, particularly in areas where epithelial integrity was weakened by absence or degradation of intercellular junctions [29]. They likewise used RM to identify that endogenous and exogenous progestins increase HIV-1 penetration into columnar epithelium and demonstrated that this effect increased the probability of virus interaction with CD4^+^ T cells and other host cell targets [30].

In a series of reports that confirmed and extended these findings, our laboratory newly defined a mechanism underlying the progestin-mediated increases in genital pathogen susceptibility, which have been long recognized in experimental infection models. Our initial studies showed that compared to mice in estrus, treating mice with DMPA or LNG reduced genital levels of the desmosomal proteins DSG1 and DSC1 and increased permeability of the vaginal epithelium to intravaginally administered low molecular mass molecules and activated leukocytes [31]. We also saw that these genital changes were DMPA dose dependent and occurred in mice with serum medroxyprogesterone acetate levels comparable to trough levels found in women using DMPA [32]. Likewise, progestin-mediated loss of genital epithelial integrity and barrier function correlated in dose-dependent fashion with enhanced susceptibility to genital herpes simplex virus type 2 (HSV-2) infection [31, 32]. Whereas mouse in diestrus (an estrus cycle stage with high levels of endogenous progesterone) and DMPA- and LNG-treated mice had thinner vaginal epithelium than mice in estrus (the estrus cycle stage with high levels of endogenous E), HSV-2 induced 25 vs. 100% mortality in mice in diestrus and progestin-treated mice, respectively [31]. These results implied that the increased epithelial permeability induced by DMPA or LNG has a more prominent role in promoting mouse HSV-2 susceptibility than progestin-mediated changes in epithelial thinning. In addition to promoting genital HSV-2 infection, we also found that treatment of wild-type mice with LNG increased susceptibility to genital *Chlamydia trachomatis* infection and treatment of humanized mice with DMPA or NET-EN induced uniform susceptibility to genital inoculation with cell-associated HIV-1 [33–35]. Interestingly, DMPA treatment of mice also enhanced genital expression of multiple kallikreins [33], serine proteases that promote degradation of desmosomal proteins [20, 21]. These results implied that DMPA-mediated changes in kallikrein protease expression promoted the lower levels of desmosomal proteins and loss of genital epithelial barrier function seen in treated animals.

Using uninfected wild-type and germ-free (gnotobiotic) mice, we also showed that DMPA-mediated increases in genital epithelial permeability induce inflammation by facilitating luminal endogenous microbiota entry into submucosal tissue [31]. These results revealed that DMPA-mediated impairment of genital epithelial barrier function occurs upstream of DMPA-mediated increases in inflammation, and provided mechanistic insight for multiple clinical investigations that reported DMPA use was associated with increased genital inflammation [36, 37]. Exploring the clinical relevance of our animal model data, we also collected ectocervical biopsies from women before and 1 month after initiating use of DMPA or LNG-releasing intrauterine system (LNG-IUS). Remarkably, these studies showed changes in tissue inflammation, DSG1 expression levels, and epithelial barrier function were analogously altered by exogenous progestins in mice and humans [31, 38]. Although none of these findings proved women using DMPA or other progestin-only contraceptives are more likely to acquire HIV, they did display an inter-study consistency that provided credible biological plausibility for prior epidemiological studies that identified DMPA as a significant risk factor for HIV acquisition [6] (Figure 1).

## Bridging the gap between basic and clinical research

The ultimate goal for policy makers, clinicians, and scientists is to provide women the resources they need to make informed contraceptive choices. Achieving this goal is decidedly complex and likely to benefit from thoughtful integration of results from diverse but complementary lines of research. Although the ECHO Trial was a well-executed study, we believe that it does not deliver any final word on the subject nor fully support the conclusion of the GDG that “women at a high risk of HIV infection are eligible to use all progestin-only contraceptive methods without restriction” [10]. In the first place, there is little data on the influence of Cu-IUD or LNG implant use on HIV susceptibility, and the ECHO Trial defined DMPA-mediated risk of HIV relative to their use. The ECHO Trial likewise did not include women randomized to use NET-EN or LNG-IUS, and it is unclear if these progestins differentially alter HIV susceptibility compared to DMPA or LNG implant. For example, it is conceivable that compared to systemic LNG release from a dermal implant, the effects of LNG on genital epithelial barrier function and HIV susceptibility are more pronounced with local release. Providing answers to these and other unresolved questions with prospective clinical studies is vital but time consuming and resource intensive. Such studies must also overcome ethical hurdles, residual confounding created by varying sexual behavior among individuals using DMPA or a long-acting reversible contraceptive vs. no contraception, and the frequency with which women discontinue or change contraceptives [7, 39]. These challenges highlight the need to utilize experimental models with the ability to acquire data unobtainable in clinical investigation, explore plausible links between HIV susceptibility and a specific contraceptive agent, and define the contraceptive choices least likely to enhance HIV transmission. To expedite our capacity to provide individuals the information needed to make informed decisions regarding contraceptive choice, it will also be useful that policymaking bodies and advisory groups become inclusive of investigators conducting basic science research and consider all pertinent and available biological data when formulating their recommendations.
